# Relationship between AQP5 and chemotherapy resistance in colorectal cancer cells and its mechanism

**DOI:** 10.1007/s12672-025-03193-9

**Published:** 2025-08-04

**Authors:** Xiaoyun Yang, Chunhua Zhou, Wencheng Kong, Tongfa Ju, Qi Xie, Xinchun Liu

**Affiliations:** 1 The Fourth School of Clinical Medicine, Zhejiang Chinese Medical University, Hangzhou First People’s Hospital, Hangzhou, 310006 China; 2 Hangzhou First People’s Hospital, Hangzhou, 310006 China

**Keywords:** Colorectal cancer, Aquaporin-5 (AQP5), Chemotherapy resistance, Apoptosis, NF-κB

## Abstract

**Background:**

Previous studies have suggested that AQP5 expression is increased in colorectal cancer tissues and is associated with the progression and prognosis of colorectal cancer. However, there are few studies on the relationship between AQP5 and chemotherapy resistance in colorectal cancer cells and the related mechanisms.

**Methods:**

In this study, AQP5 overexpression plasmid was transfected into human colorectal cancer cell lines RKO and HCT116, and the effects of AQP5 combined with 5-FU on the proliferation and apoptosis of colorectal cancer cells and the underlying mechanism were investigated by western blotting, MTT assay and flow cytometry. Then, the results of in vitro experiments were verified in vivo using SPF nude mice.

**Results:**

AQP5 overexpression plasmid transfected significantly increased AQP5 expression in colorectal cancer cell lines, MTT assay showed that AQP5 overexpression promoted the proliferation of colorectal cancer cells. 5-FU inhibited the proliferation of colorectal cancer cells. Overexpression of AQP5 can restore the inhibition of 5-FU to some extent. Flow cytometry showed that AQP5 had no significant effect on apoptosis of colorectal cancer cells. Western blotting experiments showed that the expression level of p-NF-κB protein in AQP5 overexpression group was significantly up-regulated. The results of tumor bearing experiment in nude mice (in vivo) showed that the tumor growth rate of AQP5 overexpression group was faster, and the tumor diameter and body weight of nude mice were significantly increased. After 5-FU treatment, the tumor volume became smaller, and the tumor volume in AQP5 overexpression group was significantly larger than that in control group. Immunohistochemical results showed that the expression level of p-NF-κB was up-regulated and the number of apoptosis was decreased in the 5-FU treatment group with AQP5 overexpression.

**Conclusion:**

Overexpression of AQP5 can promote the growth of colorectal cancer cells and promote the occurrence of drug resistance, which may be related to NF-κB signaling pathway. AQP5-mediated chemoresistance suggests its potential as a target for RNA-based gene silencing therapies.

**Supplementary Information:**

The online version contains supplementary material available at 10.1007/s12672-025-03193-9.

## Introduction

Colorectal cancer is one of the most common gastrointestinal malignancies, and its morbidity and mortality rank the third and second among all malignant tumors in the world [1]. With the improvement of living standards and the change of dietary habits (high fat, high protein and low dietary fiber), the incidence and mortality of colorectal cancer in China are also on the rise, which seriously threatens the life and health of the people [2]. At present, the treatment methods for colorectal cancer mainly include radical surgery, chemical therapy (referred to as “chemotherapy”), radiotherapy and molecular targeted therapy [3]. Patients with advanced colorectal cancer must rely on neoadjuvant chemotherapy or postoperative adjuvant chemotherapy to improve their survival, and chemotherapy resistance often leads to chemotherapy failure and seriously affects the prognosis of patients. Therefore, in-depth study of the mechanism of chemotherapy resistance in colorectal cancer and search for new targets for precise intervention therapy are one of the important strategies to improve the therapeutic effect of colorectal cancer.

In recent years, more and more studies have shown that tumor growth, development, invasion and metastasis depend on tumor microenvironment and metabolism [4]. Water channels play an important role in the development of tumors, and this discovery is very important for the advancement of anti-tumor therapies [5]. Aquaporins (AQPs) are membrane water transport channels involved in the secretion and absorption of epithelial cells. Some aquaporin subtypes also transport other molecules, such as glycerol and urea. In recent years, the role of AQPs in tumorigenesis and development has attracted more and more attention. Previous studies by our team have found that AQP5 expression is significantly increased in colorectal cancer tissues and 5-FU-resistant colorectal cancer cells, suggesting that AQP5 overexpression plays a certain role in colorectal tumor progression and chemotherapy resistance [6]. However, the relationship between AQP5 and chemotherapy resistance of colorectal cancer cells and the related mechanisms remain unclear. Meanwhile, chemotherapy-induced genotoxic stress triggers sustained activation of NF-κB and STAT3, with concomitant upregulation of cytokine networks that promote treatment resistance and metastatic recurrence. Our findings align with established mechanisms of chemoresistance: Long non-coding RNA CASC9 drives gemcitabine resistance via NRF2/NF-κB reciprocal amplification loops [7],and Biglycan (BGN) overexpression induces increased NF-κB pathway activity and reduced expression of pro-apoptotic markers in drug resistance experiments [8]. These findings seem to suggest that NF-κB plays an indispensable role in drug resistance. Therefore, in this study, we transfected AQP5 overexpression plasmid into human colorectal cancer cell lines RKO and HCT116 to explore the effect of AQP5 combined with 5-FU on the proliferation and apoptosis of colorectal cancer cells and the underlying mechanism, and verified the results of in vitro experiments using SPF nude mice.

## Materials and methods

### Materials

Human colorectal cancer cell line RKO cells were purchased from the cell Bank of Shanghai Chinese Academy of Sciences, and HCT116 cells were purchased from Wuhan Pricella Biological Company. AQP5 plasmid was purchased from Changsha Youbao, article number: NM_001651, 5-Fu was purchased from MCE(HY-90006). Lipofectamine 2000 was purchased from Invitrogen (11668-019), MEM medium was purchased from BI, Israel, and fetal bovine serum (FBS) was purchased from GIBCO (Grand Island, NY, USA). p-NF-κB antibodies, NF-κB antibodies and β-actin antibodies were purchased from CST. Annexin V-FITC/PI Apoptosis Detection Kit purchased from 4 A Biotech Co., Ltd. SPF naked mice, feed and bedding were purchased from Shanghai SLAC Laboratory Animal Co., LTD. Tunel kit was purchased from Wuhan Xavier Biotechnology Co., LTD. (G1212), p-NF-κB p65 antibody was purchased from Bioworld (BS4135), and HRP-Goat anti-Rabbit secondary antibody was purchased from Servicebio (GB23303).

### Methods (in vitro experiment)

#### Cell culture

RKO cells were cultured in high-glucose MEM medium containing 10% fetal bovine serum (FBS), 100 U/ml penicillin, streptomycin and 1X glutamine. HCT116 cells were cultured in high-glucose McCoy’s5A medium containing 10% fetal bovine serum (FBS), 100 U/ml penicillin and streptomycin, 1X glutamine. All cells were cultured in a humid cell incubator with 5% CO_2_ at 37℃. When the cells grew to 80%, they were passed at the ratio of 1:2 or 1:4. Pancreatic enzyme digestion and passage were performed once every 2–3 days, and cells of logarithmic growth stage were selected for subsequent experiments.

#### Cell transfection

After cell passage, cells were transfected when the 6 cm culture dish was reached 80%, 2 µl Lipofectamine 2000 was added to 50 µl Opti-MEM, gently blown and mixed, and left for 5 min. Then, the 6 µg plasmid was added to 50µM Opti-MEM, gently blown and mixed. Mix the two tubes and let them stand for 20 min. The cells were removed, the culture medium was discarded, cleaned twice with 1X PBS, and 3 ml of new culture medium was replaced. Finally, the mixture was added to the cells drop by drop, gently mixed, put into the incubator, transfected for 24–48 h for follow-up experiments.

#### MTT

After cell digestion and collection, cell count was performed to prepare cell suspension. The cells were inoculated in 96-well plates: 100 µl cell suspension was added to each well, the inoculation density was 8000 cells/well, and the edge holes were filled with sterile PBS. The 96-well plates were incubated overnight in a cell incubator, followed by the addition of the specified dose of 5-FU, 100 µl per well, with 3 multiple Wells per dose. The 96-well plates were placed in cell incubators for 24–48 h. After incubation, the culture solution was sucked up, 50 µl MTT solution was added to each well (PBS was added to form 1 mg/ml solution), incubated at 37℃ for 3 h, and then 150 µl DMSO was added to each well, which was fully dissolved and mixed in a shaker. OD values at 570 nm were measured, and Graphpad 6.02 software was used for mapping and statistics.

#### Western blotting

A total of 5 × 10^5^ cells in the logarithmic growth phase were resuspended in 0.5 mL of pre-cooled cell lysis buffer and incubated on ice for 30 min. Following centrifugation, the supernatant was collected, and protein concentration was determined. Proteins were resolved using 10% SDS-PAGE and subsequently transferred onto a nitrocellulose membrane via semi-dry transfer. The membrane was then blocked in Tris-buffered saline containing Tween 20 (TBST) with 5% skim milk. After blocking with 5% non-fat milk, the membrane was appropriately sectioned and incubated separately with primary antibodies targeting the loading control and the protein of interest at 4 °C overnight. The next day, the membrane was treated with a horseradish peroxidase-conjugated secondary antibody (dilution 1:2000; Santa Cruz) at 25 °C for 2 h. Protein bands were visualized using an enhanced chemiluminescence kit (Amersham Pharmacia Biotech, Amersham, UK). At last, images of the membrane were captured, and the results were analyzed. Western blotting images were analyzed using Quantity One software developed by Bio-Rad.

#### Flow cytometry assay for apoptosis

Cells were collected, digested with EDTA-free pancreatic enzymes, and washed with pre-cooled PBS. Dilute the binding buffer with deionized water at 1:3 (4 ml 4X binding buffer + 12 ml deionized water). The cells were suspended with 1X binding buffer and the concentration was adjusted to 1–5 × 10^6^/ml. 100 µl cell suspension was taken into a 5 ml flow tube, mixed with 5 µl Annexin V/FITC, and incubated at room temperature for 5 min away from light. Then add 10 µl 20 µg/ml Propium iodide solution (PI) and add 400 µl PBS to perform flow detection immediately. Graphpad 6.02 software was used for mapping and statistical analysis.

### Methods (in vivo experiment)

#### Stable transfer plant construction

RKO cells were cultured with MEM medium containing 10% FBS and maintained at 5% CO_2_ and 37℃. Cells with good growth status were taken and inoculated into 24-well plates at a rate of 1 × 10^4^ cells/well. After cell saturation reached 30% (within 24 h), 20 µl virus with a titer of 1.0 × 10^8^ Tu·ml^-1^ (MOI:200) was added. After 48 h, fresh culture medium was replaced and continued for 24 h. Purinomycin (final concentration 1 µg·ml^-1^) was added to each pore for screening. The culture medium containing purinomycin was changed every 24 h, and the cells were passed every 2 days. After 3 generations of screening and passing, the cells were collected and verified by Western blotting and PCR.

#### RT-PCR

Total RNA was extracted by TRizol reagent, and the RNA concentration, OD260/OD280 and OD230/OD260 ratios were measured on NanoDrop. The first cDNA strand was synthesized by RevertAid kit with 2 µg RNA. The PCR primer design is shown in the following table (Table 1). PCR reaction conditions: The first reaction at 42 °C for 1 h for cDNA synthesis, followed by denaturation at 94 °C for 5 min, and then the following 22 cycles: 94 °C 30 s, 55 °C 30 s, 72 °C 30 s. After the last cycle, the final amplification reaction was performed: 72 °C for 10 min. The butler gene β-actin was used as an internal reference. SYBR green was used for RT-PCR. Comparative Ct value method was used to analyze the experimental data. 

**Table 1:** Primer sequence table

Name	F(5’–3’)	R(5’–3’)
β-actin	AGCAGTTGTAGCTACCCGCCCA	GGCGGGCACGTTGAAGGTCT
AQP5	TGTCGGAATCTACTTCACTGGC	TCCTCGTCAGGCTCATACG

#### Subcutaneous tumor experiment

The concentration of AQP5 stably overexpressed RKO cells and their control cells was adjusted to 2.5 × 107 cells/ml under aseptic conditions. Twelve 3-week-old nude mice were randomly divided into 4 groups (3 in each group), and 0.2 ml cell suspension was inoculated subcutaneously on the back or neck of mice with a 1 ml sterile syringe. After the maximum tumor size was observed to be about 80mm3, 5-FU was administered every other day for 4 weeks, during which the weight and tumor diameter of nude mice were measured every 3 days. 4 weeks later, they complied with the principles of animal welfare and were killed by denecking. The tumor was dissected and photographed and fixed for subsequent experiments.

#### TUNEL staining

Dewaxing to water, after the slice is slightly dry, draw a small circle with a chemical pen along the outer outline of the tissue with a distance of 3–4 mm from the tissue, and wash it with pure water for 3 times after drawing, 5 min each time. Appropriate amount of tunel balancing liquid was taken according to the number of slides and the size of the tissue, and balanced for 10–30 min, then 1x balancing buffer was poured, tunel working liquid was added to the ring to cover the tissue, the slices were placed flat in a wet box, incubated at 37℃ for 2 h, and a small amount of water was added to the wet box to maintain humidity. The sections were placed in PBS (PH7.4) and washed by shaking on the decolorizing table for 3 times, 5 min each time. Blot the excess PBS in the ring, then add DAPI dye solution, and incubate at room temperature for 10 min away from light. The sections were placed in PBS (PH7.4) and washed by shaking on the decolorizing table for 3 times, 5 min each time. The slices were removed and sealed with anti-fluorescence quenching tablets. Finally, the sections were observed under a fluorescence microscope and images were collected. (DAPI ultraviolet excitation wavelength 361–389 nm, emission wavelength 420 nm, emitting blue light; FITC excites at 465–495 nm and emits at 515–555 nm, emitting green light).

#### IHC staining

BSA was applied to form a blocking ring and allowed to set at room temperature for 30 min. The blocking solution was then gently removed, and PBS mixed with the primary antibody at an appropriate dilution was added to the tissue sections. The sections were laid flat in a humidified chamber and incubated overnight at 4 °C. Following incubation, the slides were washed three times in PBS (pH 7.4) on a decolorizing shaker, each wash lasting 5 min. After gently air-drying, the sections were incubated with a secondary antibody conjugated to HRP (specific to the species of the primary antibody) at room temperature for 50 min. The slides were then washed again in PBS (pH 7.4) three times for 5 min each. Once slightly dried, freshly prepared DAB substrate solution was applied within the outlined area on the sections, and the color development was monitored under a microscope. Positive staining appeared as brown-yellow. The reaction was terminated by rinsing the sections with tap water. The sections were counterstained with resuscitation lignin for approximately 3 min, followed by washing with tap water. Hematoxylin differentiation was performed for a few seconds, then rinsed with tap water, followed by treatment with a hematoxylin blue return solution and a final rinse under running water. The sections were dehydrated, mounted, and observed under a bright-field microscope for interpretation of results.

### Statistical analysis

Each experiment was repeated at least 3 times. The data were expressed as mean ± standard deviation and analyzed by ANOVA and student t test. A P value less than 0.05 was considered statistically significant.

## Result

### AQP5 promotes malignant phenotype of colorectal cancer cells

**Fig. 1:**
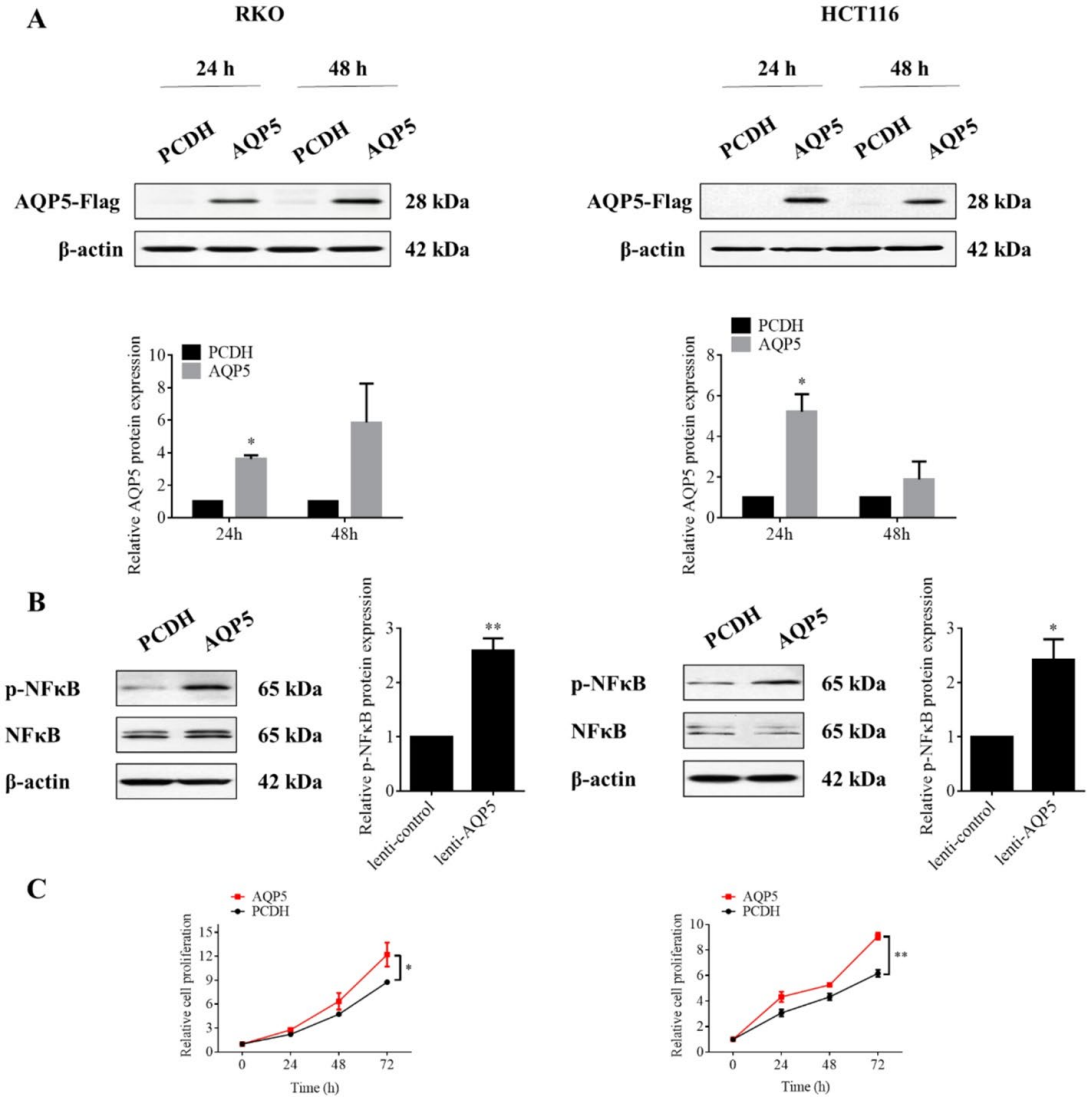
AQP5 promotes malignant phenotype of colorectal cancer cells. **A** RKO and HCT116 cells transfected AQP5 overexpression plasmid and its control plasmid for 24–48 h, and the overexpression effect was detected by Western Blotting. **B** RKO and HCT116 cells transfected AQP5 overexpression plasmid and its control plasmid for 24 h, and Western Blotting detected the related indexes of NF-κB signaling pathway. **C** RKO and HCT116 cells were transfected with AQP5 overexpression plasmid and its control plasmid for 24 h, and MTT was used to detect cell proliferation. *p < 0.05; **p < 0.01.vs. control

Firstly, to investigate AQP5’s oncogenic role, we established stable AQP5-overexpressing cell lines using colorectal cancer models RKO and HCT116. After transfecting RKO and HCT116 cells with AQP5 overexpression plasmid and its control plasmid for 24–48 h, Western Blotting confirmed successful AQP5 protein upregulation compared to empty vector controls. The results showed that AQP5 plasmid could successfully up-regulate AQP5 protein expression in RKO and HCT116 cells (Fig. 1A). MTT assays revealed significantly accelerated proliferation rates in AQP5-overexpressing groups (Fig. 1C), indicating that the overexpression of AQP5 promoted cell proliferation.

Given NF-κB's established role in proliferation and survival, we assessed pathway activation. Western Blotting experiment results showed (Fig. 1B) that compared with the control group, the expression level of p-NF-κB protein in the AQP5 overexpression group was significantly up-regulated, indicating that AQP5 overexpression markedly increased phosphorylated NF-κB levels, demonstrating functional linkage between AQP5 and NF-κB signaling.

### Effect of overexpression of AQP5 combined with 5-FU on malignant phenotype of colorectal cancer cells

**Fig. 2:**
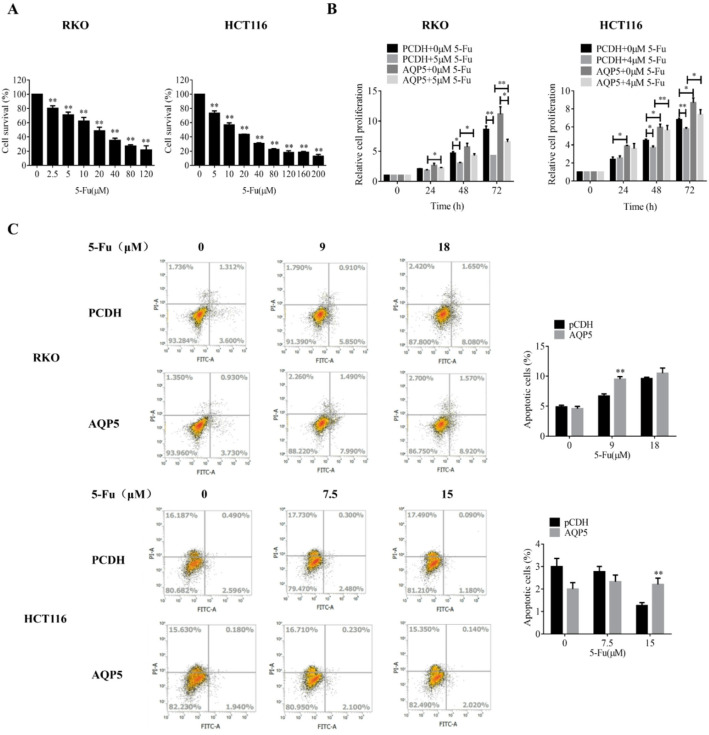
Effect of overexpression of AQP5 combined with 5-FU on malignant phenotype of colorectal cancer cells. **A** RKO and HCT116 cells were treated with 5-FU for 48 h, and the cell activity was detected by MTT. **B** RKO and HCT116 cells were transfected with AQP5 overexpression plasmid and its control plasmid for 24 h and treated with 5-FU for 48 h. MTT was used to detect cell proliferation. **C** RKO and HCT116 cells were transfected with AQP5 overexpression plasmid and its control plasmid for 24 h and treated with 5-FU for 48 h, and the apoptosis level was detected by flow cytometry. *p < 0.05; **p < 0.01.vs. control

Subsequently, we proceeded to assess the impact of 5-FU on the cell lines. To evaluate 5-FU’s therapeutic efficacy, we first determined its dose-response relationship in RKO and HCT116 cells. MTT-based cytotoxicity assays revealed concentration-dependent growth inhibition, with IC50 values of 18.55 µM for RKO cells and 15.36 µM for HCT116 cells (Fig. 2A).

Next, we examined AQP5’s role in modulating 5-FU sensitivity. Cells transfected with AQP5 overexpression or control plasmids for 24 h were treated with 5-FU for 48 h. The changes of cell proliferation and apoptosis were detected by MTT and flow cytometry. MTT assays demonstrated that 5-FU significantly suppressed proliferation, while AQP5 overexpression partially rescued this effect (Fig. 2B). Flow cytometry analysis of apoptosis showed no significant difference between AQP5-overexpressing and control groups (Fig. 2C), indicating AQP5’s primary role in proliferation regulation rather than apoptosis modulation.

### In vivo validation of AQP5 and 5-FU effects on colorectal cancer progression

**Fig. 3:**
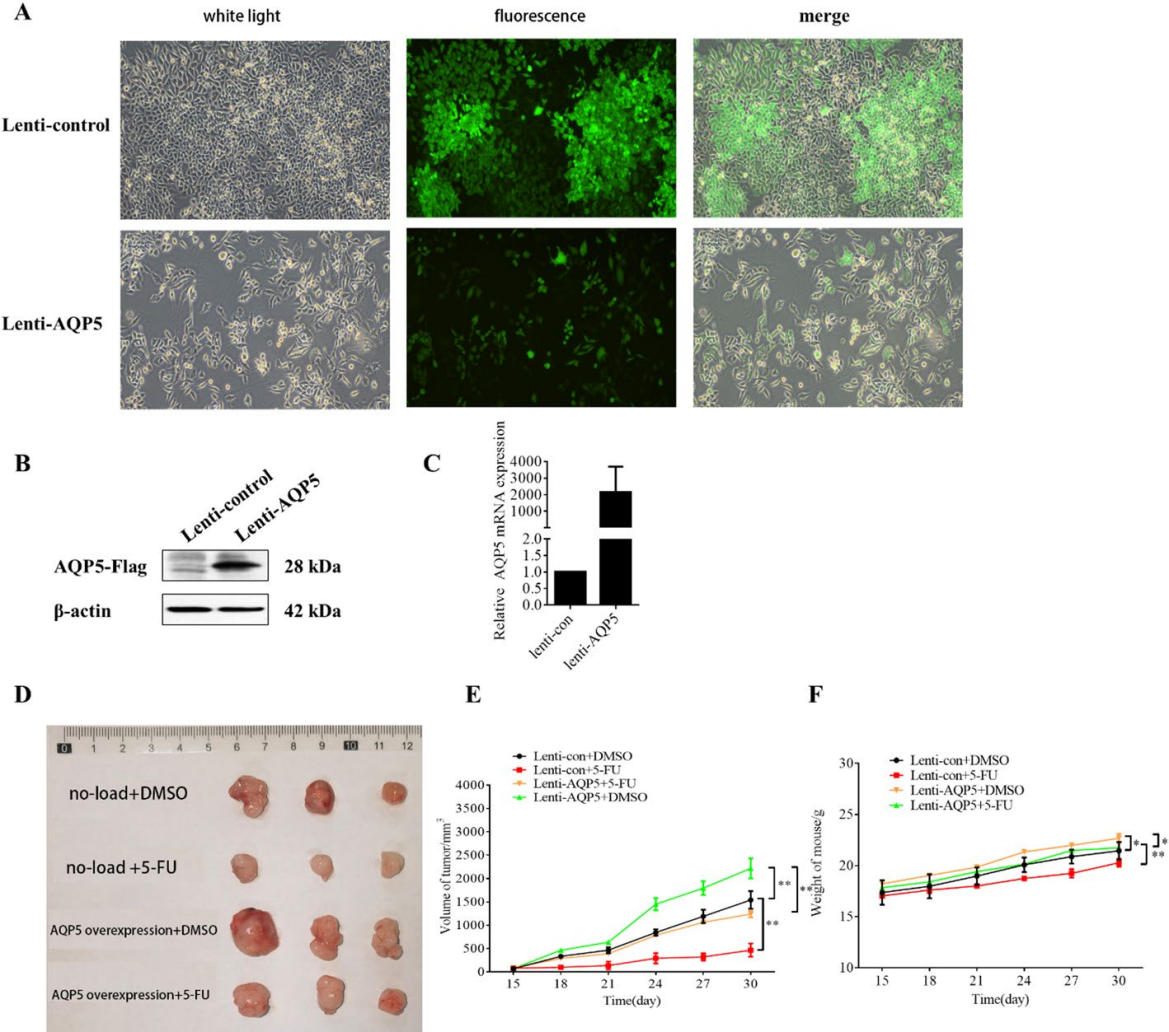
In vivo experiments confirm the effects of AQP5 and 5-FU on malignant progression of colon cancer. **A** Fluoroscopic photographs of AQP5 stable strain; **B** Western Blotting detected AQP5 expression levels in stable RKO strains. **C** PCR was used to detect AQP5 expression in stable RKO strains. **D** Overall tumor map; E: Tumor diameter map of nude mice; **F** Weight map of nude mice. *p < 0.05; **p < 0.01.vs. control

To establish an in vivo model, RKO cells were transduced with AQP5-overexpressing lentivirus. Stable AQP5 overexpression cell line was constructed, and cell fluorescence was observed under an inverted microscope. When the proportion of green fluorescence in the cells reached about 90%, total protein and total RNA were extracted from part of the cells, and the effect of AQP5 overexpression was verified by Western Blotting and PCR. The results showed that AQP5 expression levels in RKO cells were successfully up-regulated (Fig. 3A–C).

Next, different cell suspensions were injected into the backs or necks of the mice, resulting in distinct tumor growth patterns. The subcutaneous tumor bearing experiment (Fig. 3D–F) showed that AQP5-overexpressing tumors exhibited accelerated growth rates, accompanied by increased tumor diameter and elevated mouse body weight, indicating that overexpression of AQP5 can promote the growth of colorectal cancer tumors. Notably, AQP5 overexpression partially reversed 5-FU’s therapeutic effects. Compared to the no-load + 5FU group, AQP5-overexpressing tumors showed increased diameter and body weight, demonstrating AQP5-mediated chemoresistance.

### Tumor histopathological analysis reveals AQP5-mediated NF-κB activation

**Fig. 4:**
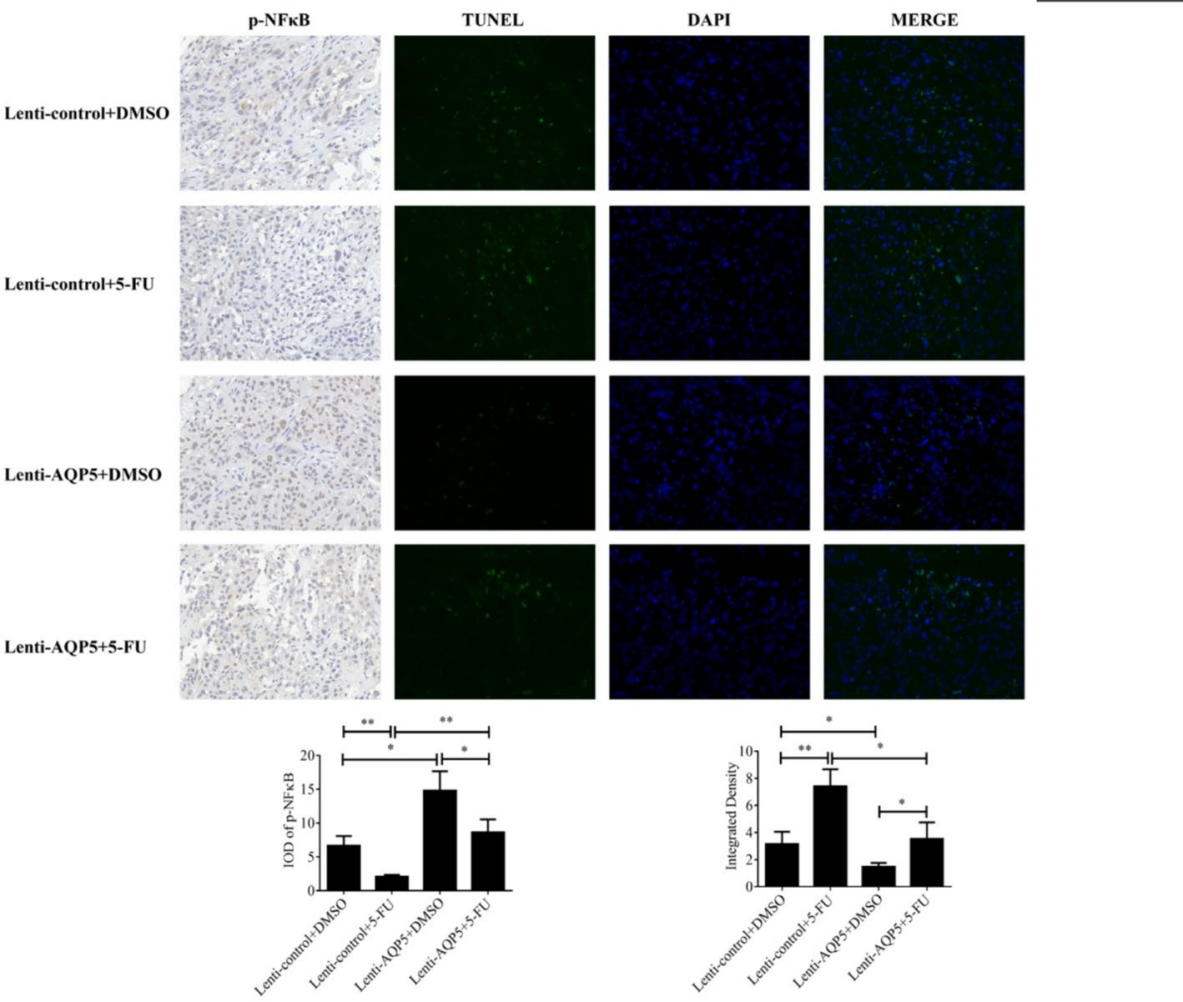
Immunohistochemical and TUNEL results of subcutaneous graft tumors. p-NF-κB was detected by immunohistochemistry of subcutaneous graft tumor and apoptosis level was detected by TUNEL (200×). *p < 0.05; **p < 0.01.vs. control

Finally, we prepared sections of the tumor tissue and observed them under a fluorescence microscope. Immunohistochemical analysis of tumor sections (Fig. 4) demonstrated significant p-NF-κB upregulation in AQP5-overexpressing tumors compared to controls, accompanied by reduced apoptotic cells.

Crucially, AQP5 overexpression counteracted 5-FU’s therapeutic effects: compared with the group treated with 5-FU alone, the expression level of p-NF-κB was up-regulated and the number of apoptosis was reduced in the group treated with AQP5 overexpression and 5-FU alone, suggesting that AQP5 could restore the inhibitory effect of 5-FU on the malignant progression of colorectal cancer.

## Discussion

Currently, thirteen aquaporin (AQP) species have been identified in mammals, which are broadly categorized into three main groups [9, 10]: (1) Classical AQPs, as primarily water-selective channels, including AQP0, AQP1, AQP2, AQP4, AQP5, AQP6 and AQP8; (2) Aquaglyceroporins, such as AQP3, AQP7, AQP9 and AQP10, which facilitate the transport of glycerol and other small solutes in addition to water; and (3) Non-classical AQPs, comprising AQP11 and AQP12, which are localized intracellularly but whose substrate specificity remains unclear. As the name implies, AQPs are specialized channels that facilitate the efficient and selective transport of water molecules. They are widely distributed in the cell membranes of both prokaryotic and eukaryotic organisms [11]. As is known to all, AQPs are responsible for maintaining water homeostasis and regulating multiple physiological and pathological processes [12]. The development of tumors arises from the disruption of normal cell growth regulation at the genetic level, driven by various tumorigenic factors. Meanwhile, tumor progression relies on multiple metabolic processes that involve water molecules, with AQPs facilitating their rapid and selective transport [13]. Previous studies have demonstrated that AQPs are highly expressed in various tumor cells and are associated with tumor-related edema, cell migration, proliferation, and angiogenesis [14, 15]. For instance, the occurrence of brain tumors is often associated with the expression of AQP1 [16]. And mice deficient in AQP3 exhibit a strong resistance to skin tumors [17].

AQP5, a 21-24kda protein, serves as a key component of cell membrane vesicles. It plays a role in tissue carcinogenic transformation and malignant tumor progression [18, 19]. Researches have shown that elevated AQP5 expression is associated with tumor lymphatic metastasis and poor prognosis, which is observed in breast cancer [20], gastric cancer [21], liver cancer [22], lung cancer [23] and cervical cancer [24]. Additionally, AQP5 expression is upregulated in colorectal cancer tissues and influences colorectal cancer prognosis [25] by modulating extracellular regulated protein kinase-1/2(ERK1/2) [26] and p38 MAPK signaling pathway [27]. Other studies have revealed that silencing AQP5 expression can promote apoptosis of colorectal tumor cells, and AQP5 expression is related to not only higher TNM stage, lymph node metastasis and distant metastasis, but also the number of peripheral blood circulating tumor cells (CTC) of patients [25]. However, the specific mechanism by which AQP5 promotes tumor development remains unclear. Previous studies have shown that high expression of APQ5 can promote cell proliferation, inhibit cell apoptosis, reset cell cycle, and promote epithelial mesenchymal transformation and cell migration [28]. Aqp5-induced changes in tumor biological behavior may be regulated by a variety of signaling pathways leading to cell transformation, including tyrosine kinase Src family, epidermal growth factor receptor, Wnt signaling pathway and Erk1/2 mediated signal transduction [28–30].

The NF-κB signaling pathway, as a crucial cellular signaling pathway, is extensively involved in biological processes such as immune responses, inflammatory reactions, cell proliferation, survival, and apoptosis. Generally, the NF-κB pathway is divided into the canonical pathway and the non-canonical pathway, each with distinct activation mechanisms. The canonical NF-κB pathway is rapid and transient. Upon stimulation by extracellular signals such as TNF and IL-1, the NF-κB pathway activates IκB kinase complex (IKK), which subsequently leads to the phosphorylation, ubiquitination, and proteasomal degradation of IκB. These result in the release and nuclear translocation of the p65/p50 heterodimers, thereby activating downstream anti-apoptotic genes such as BCL-XL and BCL2, thus promoting tumor cell survival and tumor progression. Additionally, the canonical NF-κB pathway also promotes angiogenesis by regulating pro-angiogenic genes such as vascular endothelial growth factor (VEGF) and CXC chemokine ligand 8 (CXCL8), facilitating tumor invasion. Furthermore, NF-κB can promote their lymphatic and hematogenous metastasis by inducing epithelial-to-mesenchymal transition (EMT). In contrast, the activation of the non-canonical pathway is relatively slow and is often induced by the TNF receptor (TNFR) superfamily. Under the mediation of NF-κB-inducing kinase (NIK) and IKK, the RelB/p52 complex translocates to the nucleus to activate target genes [31]. Further investigation reveals that NF-κB can upregulate the expression of multidrug resistance proteins (such as MDR1/P-gp), increasing drug efflux and reducing intracellular drug concentrations, thereby diminishing the efficacy of chemotherapeutic agents [32, 33]. NF-κB alters the tumor microenvironment by modulating the secretion of cytokines and chemokines (GDF15, CCL3, and CCL4), promoting tumor cell survival and drug resistance [34].

Our present findings suggest that AQP5 overexpression can promote colorectal cancer cell proliferation, and the mechanism may be related to activation of the NF-κB signaling pathway. Overexpression of AQP5 has a certain recovery effect on the proliferation of colorectal cancer cells inhibited by 5-Fu. In vivo experiments confirmed that overexpression of AQP5 can promote the growth of colon cancer tumor, and overexpression of AQP5 can restore the inhibition of 5-Fu on the progression of colorectal cancer. Interestingly, our in vitro experiments found that AQP5 seemed to have no effect on apoptosis of colorectal cancer cells (Negative result), but animal experiments found that overexpression of AQP5 could reduce apoptosis of colorectal cancer cells. The reasons for this contradictory result may be related to the following two points: (1) the difference between in vitro and in vivo environment: In vitro experiment is carried out in a cell culture dish, and the cells are in a relatively simple environment, lacking the regulation of complex physiological, biochemical and immune factors in vivo. In vivo experiments (animal experiments) can more truly simulate the physiological environment of the human body, including the interaction between cells and the influence of humoral factors. Therefore, the results of in vitro experiments may not fully reflect the situation in vivo. (2) Differences in detection methods: In this study, flow cytometry was used to detect apoptosis in vitro, while TUNEL method was used in vivo. The sensitivity and specificity of the two methods may be different, resulting in differences in experimental results.

In summary, overexpression of AQP5 can promote the growth of colorectal cancer tumor and promote the occurrence of chemotherapy resistance, and the mechanism may be related to NF-κB signaling pathway. Recent studies have revealed the therapeutic potential of small RNA molecules such as microRNAs (miRNAs) in overcoming chemotherapy resistance. Notably, our previous research has demonstrated that miR-185-3p enhances the chemosensitivity of colorectal cancer cells by targeting AQP5 [6], suggesting that AQP5 may serve as a regulatory target of miRNA-based therapeutics. Given that AQP5-mediated activation of the NF-κB pathway contributes to chemoresistance, miRNAs that suppress AQP5 expression or downstream NF-κB signaling could represent a novel therapeutic approach. This is consistent with emerging evidence in other diseases, such as preeclampsia, where miRNAs such as miR-510-3p effectively regulate disease progression by targeting VEGFA and related pathways [35]. Therefore, future research should focus on identifying miRNAs that modulate the AQP5/NF-κB axis in colorectal cancer, with the aim of developing small RNA-based therapeutics to reverse chemotherapy resistance and improve treatment outcomes.

## Electronic supplementary material

Supplementary Material 1

## Data Availability

The datasets generated during and/or analysed during the current study are available from the corresponding author on reasonable request.

## References

[1] Bray F, Laversanne M, Sung H, Ferlay J, Siegel RL, Soerjomataram I, Jemal A. Global cancer statistics 2022: GLOBOCAN estimates of incidence and mortality worldwide for 36 cancers in 185 countries. CA Cancer J Clin. 2024;74(3):229–63. 38572751 10.3322/caac.21834

[2] Xia C, Dong X, Li H, Cao M, Sun D, He S, Yang F, Yan X, Zhang S, Li N, Chen W. Cancer statistics in China and united states, 2022: profiles, trends, and determinants. Chin Med J (Engl). 2022;135(5):584–90. 35143424 10.1097/CM9.0000000000002108 PMC8920425

[3] Favoriti P, Carbone G, Greco M, Pirozzi F, Pirozzi RE, Corcione F. Worldwide burden of colorectal cancer: a review. Updates Surg. 2016;68(1):7–11. 27067591 10.1007/s13304-016-0359-y

[4] Koontongkaew S. The tumor microenvironment contribution to development, growth, invasion and metastasis of head and neck squamous cell carcinomas. J Cancer. 2013;4(1):66–83. 23386906 10.7150/jca.5112 PMC3564248

[5] Sekine S, Shimada Y, Nagata T, Moriyama M, Omura T, Watanabe T, Hori R, Yoshioka I, Okumura T, Sawada S, Fukuoka J, Tsukada K. Prognostic significance of Aquaporins in human biliary tract carcinoma. Oncol Rep. 2012;27(6):1741–7. 22470085 10.3892/or.2012.1747

[6] Zhou C, Kong W, Ju T, Xie Q, Zhai L. MiR-185-3p mimic promotes the chemosensitivity of CRC cells via AQP5. Cancer Biol Ther. 2020;21(9):790–8. 32588739 10.1080/15384047.2020.1761238 PMC7515541

[7] Zhang Z, Chen L, Zhao C, Gong Q, Tang Z, Li H, Tao J. CASC9 potentiates gemcitabine resistance in pancreatic cancer by reciprocally activating NRF2 and the NF-κB signaling pathway. Cell Biol Toxicol. 2023;39(4):1549–60. 35913601 10.1007/s10565-022-09746-w

[8] Liu B, Xu T, Xu X, Cui Y, Xing X. Biglycan promotes the chemotherapy resistance of colon cancer by activating NF-κB signal transduction. Mol Cell Biochem. 2018;449(1–2): 285– 94. 10.1007/s11010-018-3365-1 29761248

[9] Ishibashi K, Tanaka Y, Morishita Y. The role of mammalian superaquaporins inside the cell. Biochim Biophys Acta. 2014;1840(5):1507–12. 24189537 10.1016/j.bbagen.2013.10.039

[10] Benga G. On the definition, nomenclature and classification of water channel proteins (aquaporins and relatives). Mol Aspects Med. 2012;33(5–6):514–7. 22542572 10.1016/j.mam.2012.04.003

[11] Abascal F, Irisarri I, Zardoya R. Diversity and evolution of membrane intrinsic proteins. Biochim Biophys Acta. 2014;1840(5):1468–81. 24355433 10.1016/j.bbagen.2013.12.001

[12] McCoy E, Sontheimer H. Expression and function of water channels (aquaporins) in migrating malignant astrocytes. Glia. 2007;55(10):1034–43. 17549682 10.1002/glia.20524 PMC2561225

[13] Ribatti D, Ranieri G, Annese T, Nico B. Aquaporins in cancer. Biochim Biophys Acta. 2014;1840(5):1550–3. 24064112 10.1016/j.bbagen.2013.09.025

[14] Verkman AS, Hara-Chikuma M, Papadopoulos MC. Aquaporins–new players in cancer biology. J Mol Med (Berl). 2008;86(5):523–9. 18311471 10.1007/s00109-008-0303-9 PMC3590015

[15] Papadopoulos MC, Saadoun S. Key roles of Aquaporins in tumor biology. Biochim Biophys Acta. 2015;1848(10 Pt B):2576–83. 25204262 10.1016/j.bbamem.2014.09.001

[16] Wang D, Owler BK. Expression of AQP1 and AQP4 in paediatric brain tumours. J Clin Neurosci. 2011;18(1):122–7. 20965731 10.1016/j.jocn.2010.07.115

[17] Hara-Chikuma M, Verkman AS. Prevention of skin tumorigenesis and impairment of epidermal cell proliferation by targeted aquaporin-3 gene disruption. Mol Cell Biol. 2008;28(1):326–32. 17967887 10.1128/MCB.01482-07 PMC2223314

[18] Zhang Z, Chen Z, Song Y, Zhang P, Hu J, Bai C. Expression of Aquaporin 5 increases proliferation and metastasis potential of lung cancer. J Pathol. 2010;221(2):210–20. 20455256 10.1002/path.2702

[19] Jung HJ, Park JY, Jeon HS, Kwon TH. Aquaporin-5: a marker protein for proliferation and migration of human breast cancer cells. PLoS ONE. 2011;6(12):e28492. 22145049 10.1371/journal.pone.0028492 PMC3228775

[20] Lee SJ, Chae YS, Kim JG, Kim WW, Jung JH, Park HY, Jeong JY, Park JY, Jung HJ, Kwon TH. AQP5 expression predicts survival in patients with early breast cancer. Ann Surg Oncol. 2014;21(2):375–83. 24114055 10.1245/s10434-013-3317-7

[21] Shen L, Zhu Z, Huang Y, Shu Y, Sun M, Xu H, Zhang G, Guo R, Wei W, Wu W. Expression profile of multiple Aquaporins in human gastric carcinoma and its clinical significance. Biomed Pharmacother. 2010;64(5):313–8. 20106632 10.1016/j.biopha.2009.12.003

[22] Guo X, Sun T, Yang M, Li Z, Li Z, Gao Y. Prognostic value of combined Aquaporin 3 and Aquaporin 5 overexpression in hepatocellular carcinoma. Biomed Res Int. 2013;2013:206525. 24224160 10.1155/2013/206525 PMC3810059

[23] Chae YK, Woo J, Kim MJ, Kang SK, Kim MS, Lee J, Lee SK, Gong G, Kim YH, Soria JC, Jang SJ, Sidransky D, Moon C. Expression of Aquaporin 5 (AQP5) promotes tumor invasion in human Non small cell lung cancer. PLoS ONE. 2008;3(5):e2162. 18478076 10.1371/journal.pone.0002162 PMC2364652

[24] Zhang T, Zhao C, Chen D, Zhou Z. Overexpression of AQP5 in cervical cancer: correlation with clinicopathological features and prognosis. Med Oncol. 2012;29(3):1998–2004. 22048942 10.1007/s12032-011-0095-6

[25] Shan T, Cui X, Li W, Lin W, Li Y. AQP5: a novel biomarker that predicts poor clinical outcome in colorectal cancer. Oncol Rep. 2014;32(4):1564–70. 25109507 10.3892/or.2014.3377

[26] Woo J, Lee J, Chae YK, Kim MS, Baek JH, Park JC, Park MJ, Smith IM, Trink B, Ratovitski E, Lee T, Park B, Jang SJ, Soria JC, Califano JA, Sidransky D, Moon C. Overexpression of AQP5, a putative oncogene, promotes cell growth and transformation. Cancer Lett. 2008;264(1):54–62. 18423983 10.1016/j.canlet.2008.01.029 PMC3074481

[27] Shi X, Wu S, Yang Y, Tang L, Wang Y, Dong J, Lü B, Jiang G, Zhao W. AQP5 Silencing suppresses p38 MAPK signaling and improves drug resistance in colon cancer cells. Tumour Biol. 2014;35(7):7035–45. 24752576 10.1007/s13277-014-1956-3

[28] Direito I, Madeira A, Brito MA, Soveral G. Aquaporin-5: from structure to function and dysfunction in cancer. Cell Mol Life Sci. 2016;73(8):1623–40. 26837927 10.1007/s00018-016-2142-0 PMC11108570

[29] Woo J, Lee J, Kim MS, Jang SJ, Sidransky D, Moon C. The effect of Aquaporin 5 overexpression on the Ras signaling pathway. Biochem Biophys Res Commun. 2008;367(2):291–8. 18155156 10.1016/j.bbrc.2007.12.073

[30] Choi JH, Wu HG, Jung KC, Lee SH, Kwon EK. Apoptosis and expression of AQP5 and TGF-beta in the irradiated rat submandibular gland. Cancer Res Treat. 2009;41(3):145–54. 19809564 10.4143/crt.2009.41.3.145 PMC2757666

[31] Struzik J, Szulc-Dąbrowska L. NF-κB signaling in targeting tumor cells by oncolytic viruses-therapeutic perspectives. Cancers. 2018;10(11). 10.3390/cancers10110426 PMC6265863 30413032

[32] Bentires-Alj M, Barbu V, Fillet M, Chariot A, Relic B, Jacobs N, Gielen J, Merville MP, Bours V. NF-kappaB transcription factor induces drug resistance through MDR1 expression in cancer cells. Oncogene. 2003;22(1):90–7. 12527911 10.1038/sj.onc.1206056

[33] Kim HG, Hien TT, Han EH, Hwang YP, Choi JH, Kang KW, Kwon KI, Kim BH, Kim SK, Song GY, Jeong TC, Jeong HG. Metformin inhibits P-glycoprotein expression via the NF-κB pathway and CRE transcriptional activity through AMPK activation. Br J Pharmacol. 2011;162(5):1096–108. 21054339 10.1111/j.1476-5381.2010.01101.x PMC3051382

[34] Chen YL, Tang C, Zhang MY, Huang WL, Xu Y, Sun HY, Yang F, Song LL, Wang H, Mu LL, Li MH, Zheng WW, Miao Y, Ding LX, Li BS, Shen SH, Liu SL, Li H, Zhu ZQ, Chen HW, Tang ZH, Chen J, Hong DL, Chen HZ, Duan CW, Zhou BS. Blocking ATM-dependent NF-κB pathway overcomes niche protection and improves chemotherapy response in acute lymphoblastic leukemia. Leukemia. 2019;33(10):2365–78. 30940905 10.1038/s41375-019-0458-0

[35] Selvakumar SC, Preethi KA, Sekar D. MicroRNA-510-3p regulated vascular dysfunction in preeclampsia by targeting vascular endothelial growth factor A (VEGFA) and its signaling axis. Placenta. 2024;153:31–52. 38820941 10.1016/j.placenta.2024.05.135

