# Supplementary material for: Relationship between AQP5 and chemotherapy resistance in colorectal cancer cells and its mechanism
Source: Discov Oncol. 2025 Aug 4;16:1468. doi: 10.1007/s12672-025-03193-9 (PMC12321721; doi:10.1007/s12672-025-03193-9)

Figure 1A. RKO and HCT116 cells transfected AQP5 overexpression plasmid and its control plasmid for 24 or 48h, and the overexpression effect was detected by Western Blotting

RKO

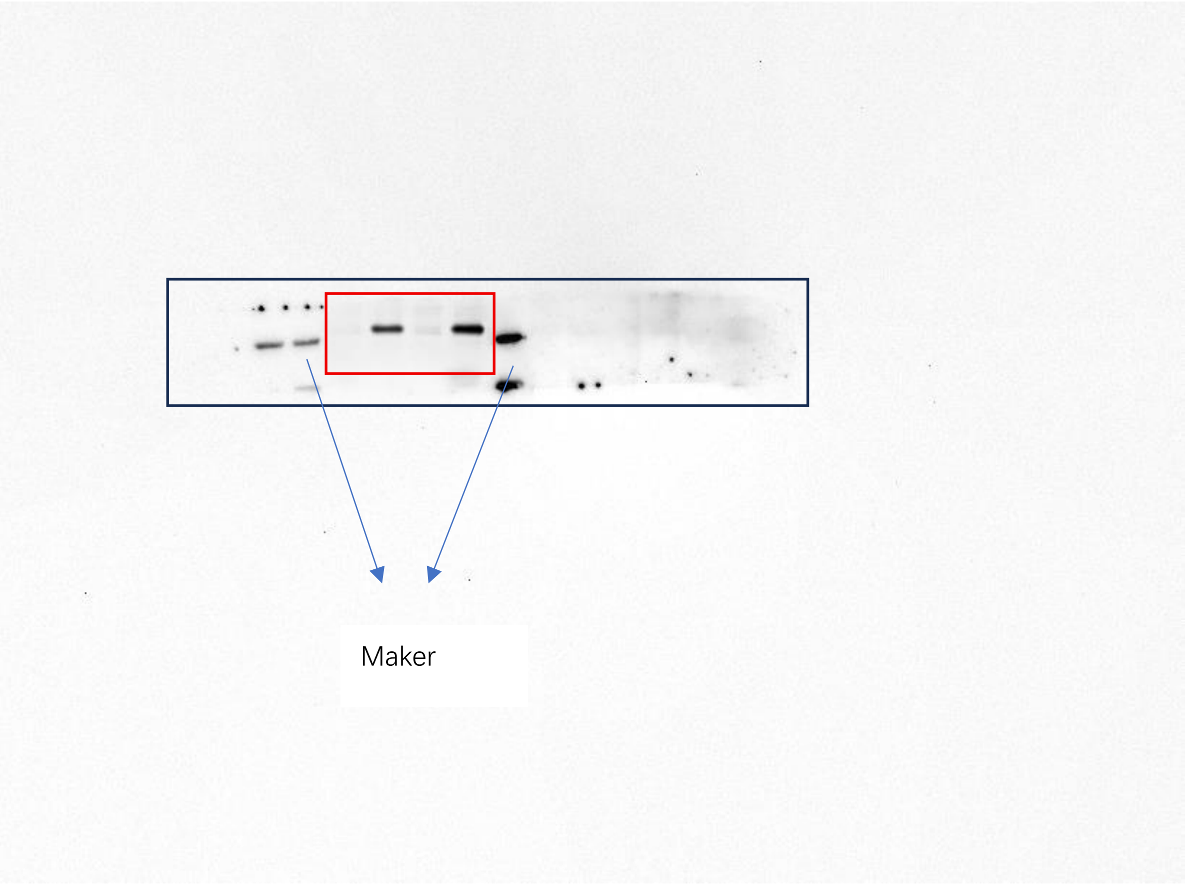

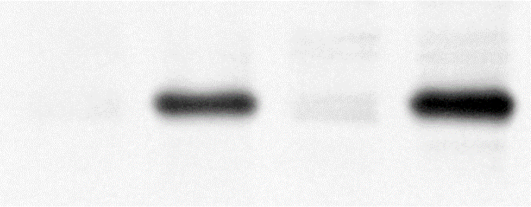

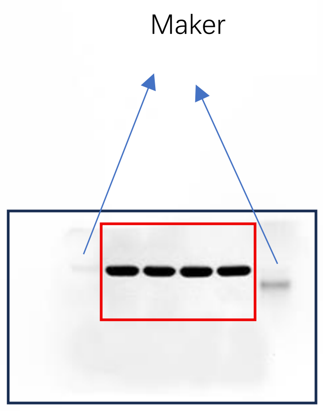
Maker

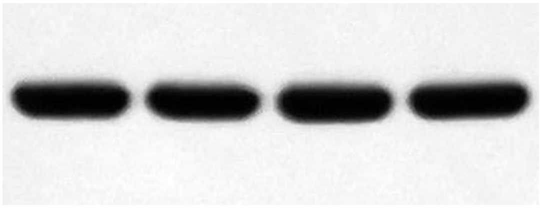

HCT116

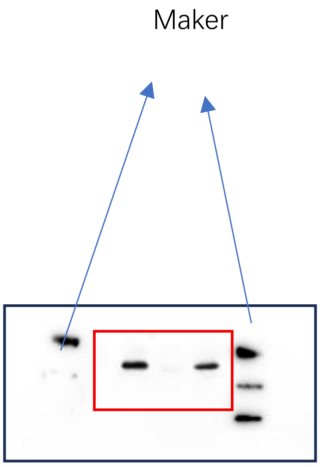

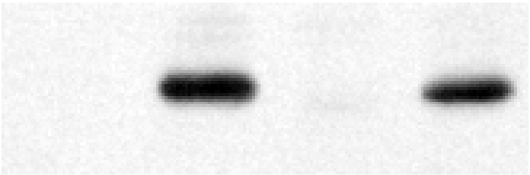

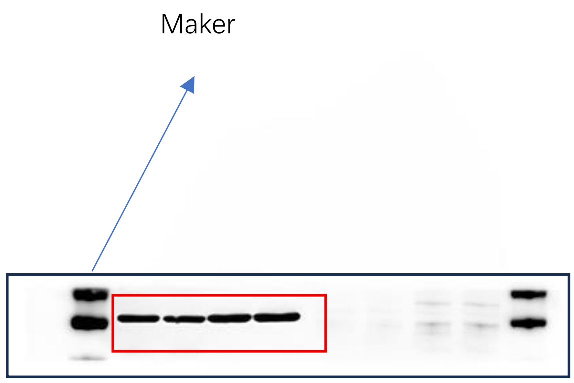
Maker

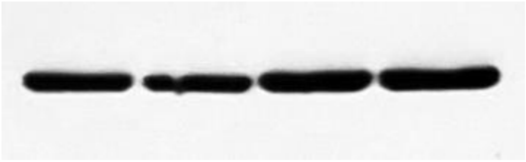

Figure 1B. RKO and HCT116 cells transfected AQP5 overexpression plasmid and its control plasmid for 24 h, and Western Blotting detected the related indexes of NF-κB signaling pathway.

RKO

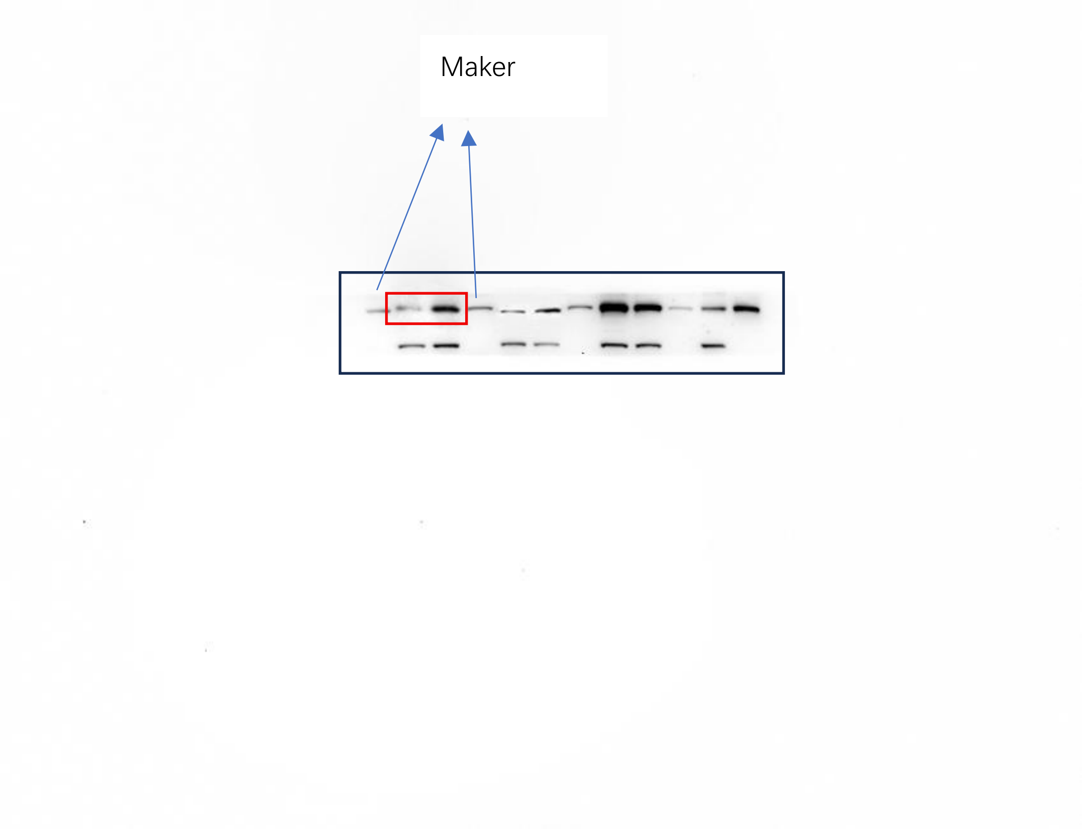

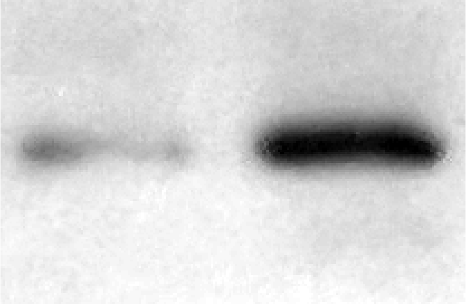

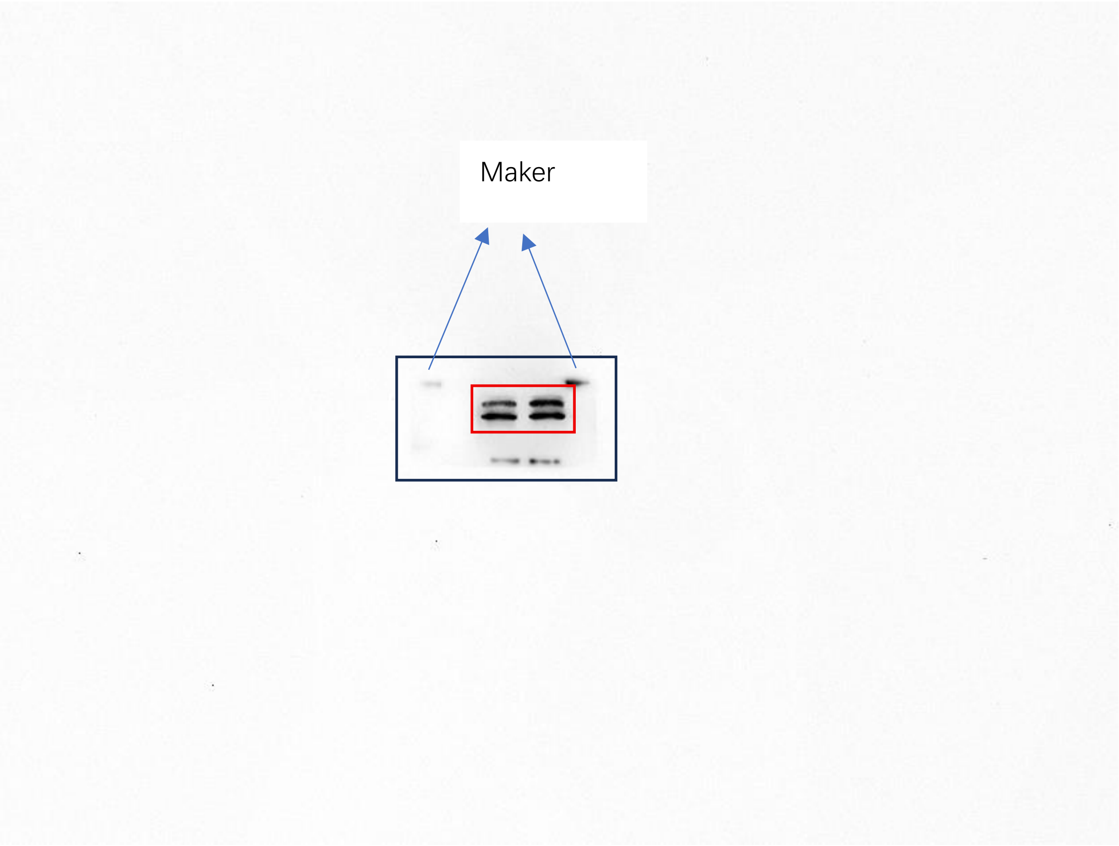
Maker

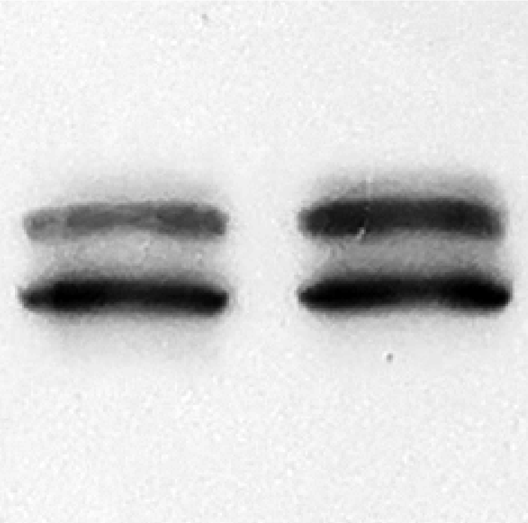

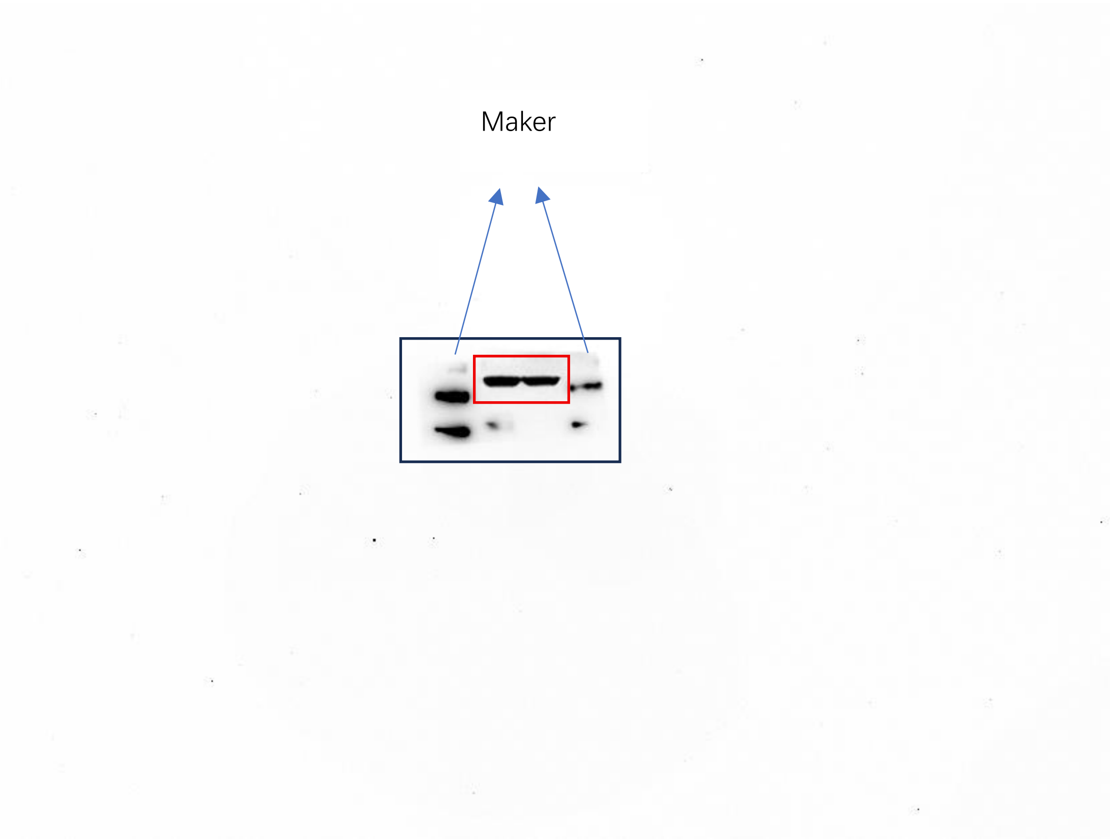
Maker

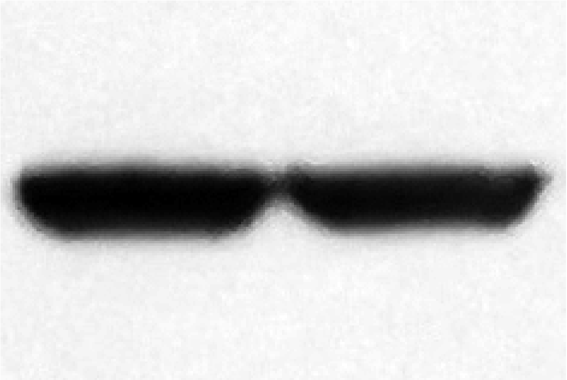

HCT116

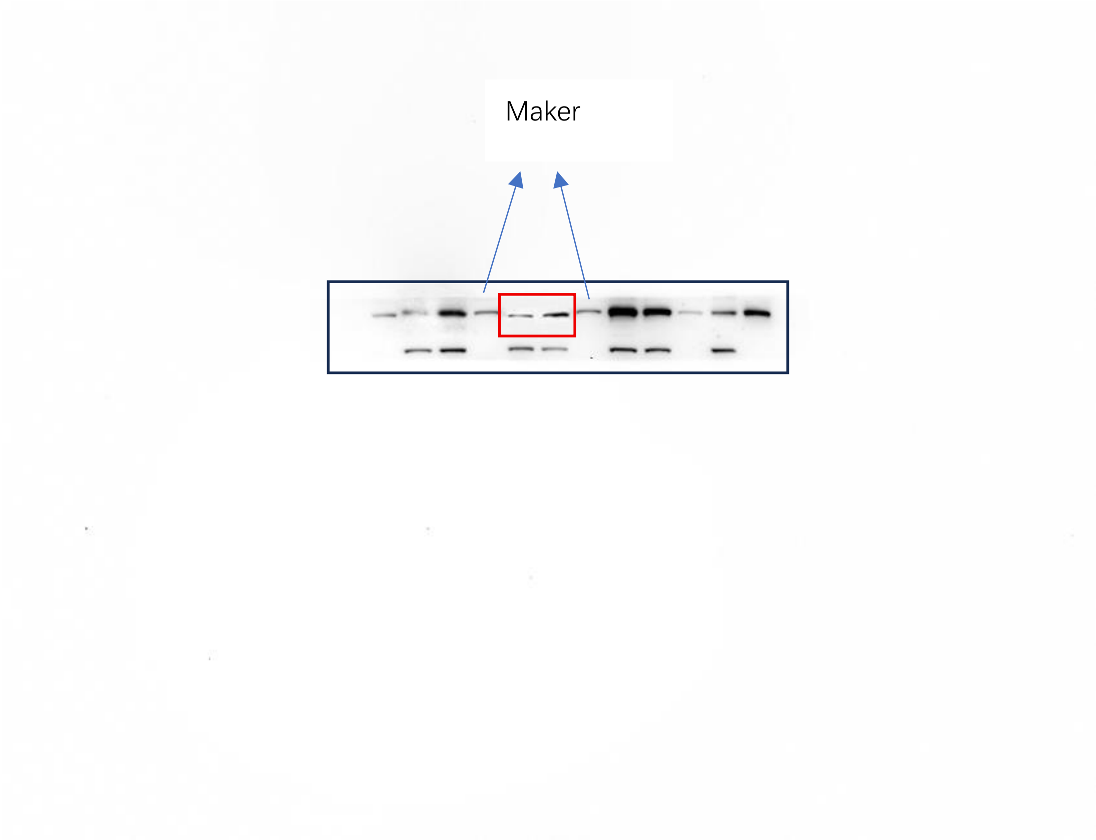

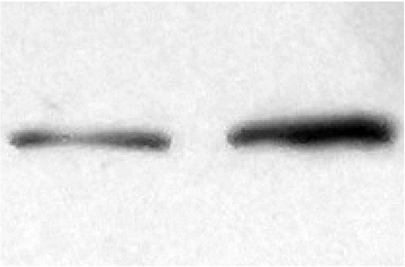

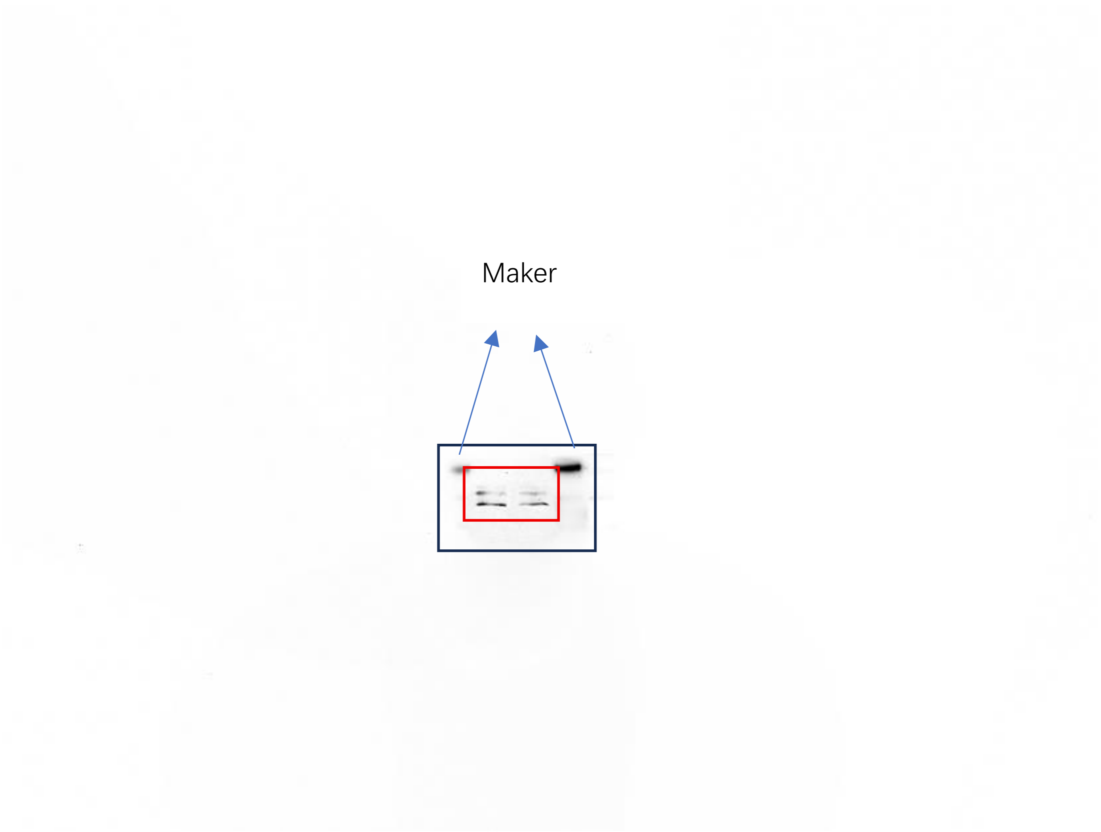
Maker

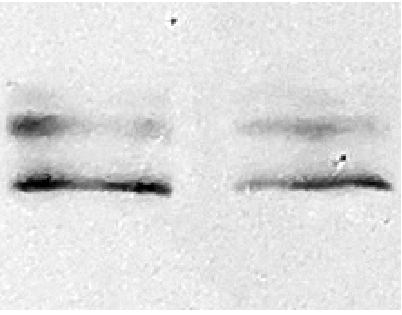

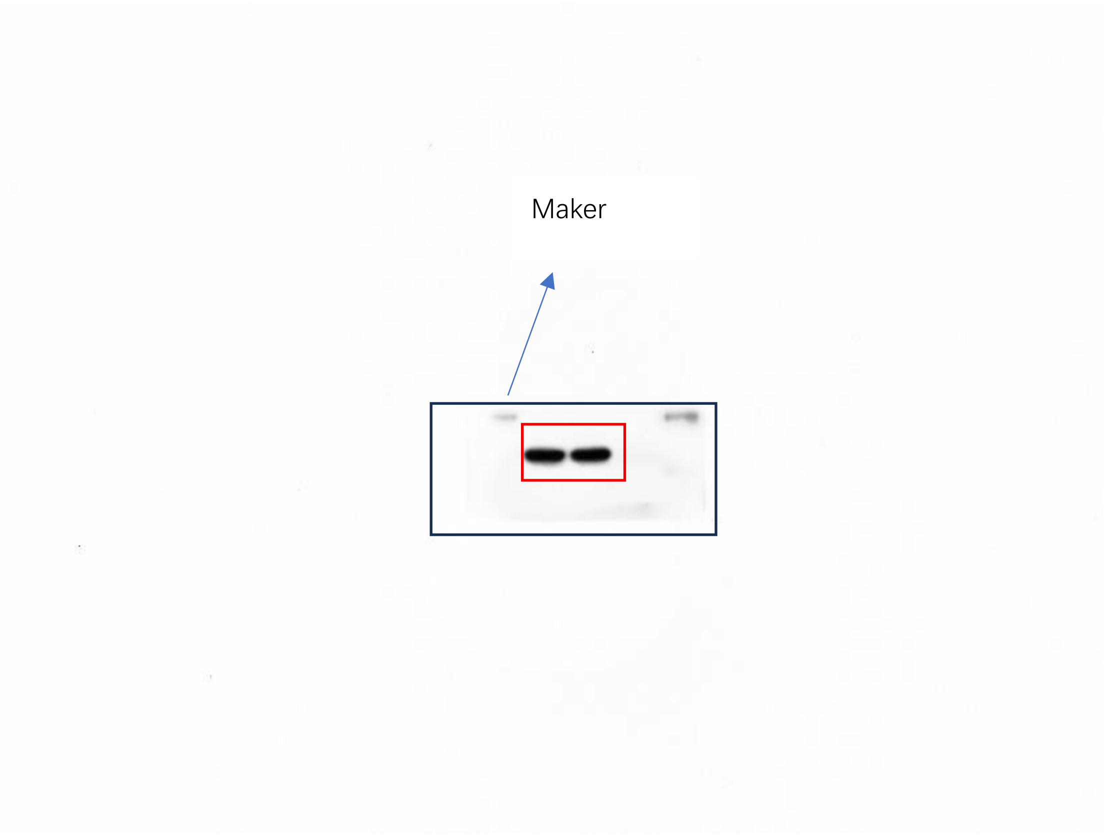
Maker

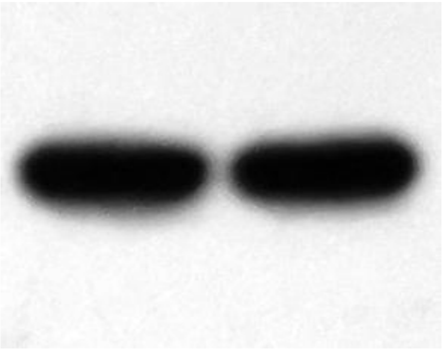

Figure 3B. Western Blotting detected AQP5 expression levels in stable RKO strains

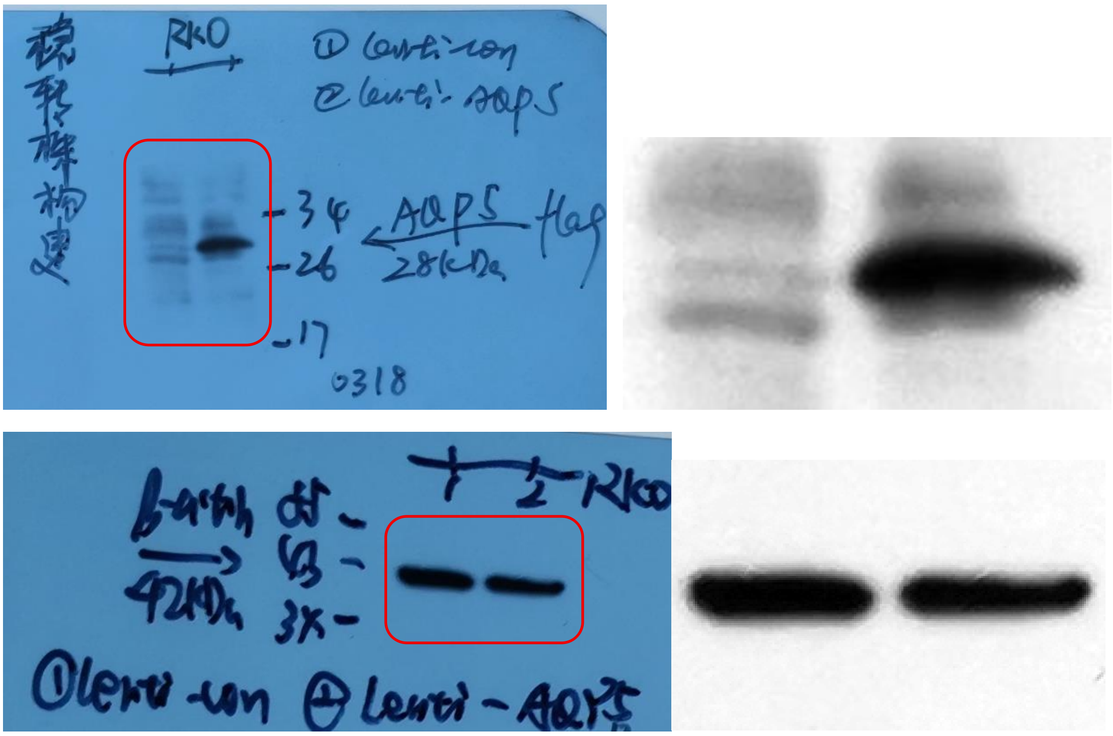

Supplement: Supplementary file 1 — Supplementary Material 1 [file 12672_2025_3193_MOESM1_ESM.pdf]
